# Freehand Transperineal Prostate Biopsy Improves the Detection Rate of Clinically Significant Prostate Cancer

**DOI:** 10.5152/tud.2025.24026

**Published:** 2025-03-06

**Authors:** Juan Manuel Bujaldon, Gonzalo Juan Vitagliano, Leandro Blas, Martin Maqueda Vocos, Hernando Rios Pita

**Affiliations:** 1 Institute of Hospital Aleman, Buenos Aires, Argentina

**Keywords:** Prostatic neoplasms, transperineal biopsy, freehand

## Abstract

**Objective:**

Freehand transperineal prostate biopsy (fTP-Bx) has been established as an alternative within the transperineal approach for prostate cancer (CaP) diagnosis. The primary objective was to compare the rate of overall CaP detection and clinically significant (greater than or equal to International Society of Urological Pathology 2) in patients with biopsy-naive between freehand transperineal prostate biopsy (fTP-Bx) and transperineal grid biopsy (TP-Bx) techniques. The secondary objective was to show the characteristics of the procedure and measure the associated complications.

**Methods:**

A retrospective review of all patients who underwent fTP-Bx (n: 326) and TP-Bx (n: 118) in our department was conducted between October 2020 and May 2023 due to suspected CaP based on elevated prostate-specific antigen (PSA), suspicious digital rectal examination, or those under active surveillance protocol. The chi-square test and Fisher’s exact test were used to compare categorical variables.

**Results:**

The mean age of the total cohort was 67.5 years (standard deviation 8.8). The overall CaP detection rate in the entire population was 70.1% (315/444), 73.9% for fTP-Bx and 62.7% for TP-Bx (*P* = .0021). The detection rate of clinically significant CaP in biopsy-naive patients was 59.7% for fTP-Bx and 45.5% for TP-Bx (*P* = .019). No patient required hospitalization for sepsis or had a positive culture-confirmed infection in the fTP-Bx group.

**Conclusion:**

Freehand transperineal prostate biopsy achieved a higher rate of clinically significant CaP detection than TP-Bx in biopsy-naive patients, accompanied by a lower number of postoperative complications.

**Main Points**

- Freehand refers to not using any device other than the needle and transducer separately.
- There were a statistically significant difference in favor of fTP-Bx regarding the detection rate of clinically significant cancer in the Bx-Naive group.
- There were no cases of urinary infection confirmed by urine culture or the need for hospitalization due to sepsis in the fTP-Bx cohort.

## Introduction

Recently, transperineal approach (TPA) has gained increasing popularity due to comparable cancer detection rates,^1-3^ reduced risk of sepsis, and improved antibiotic stewardship relative to the transrectal approach.^4-6^ Transperineal approach biopsies show an almost complete absence of post-biopsy sepsis.^7^

Although the TPA is safe and effective, it is also know that it has its disadvantages. The most important of these are the greater risk of acute urinary retention (up to 25%).^8^ and the need for general anesthesia (although in the literature, series have already been reported as a procedure under local anesthesia), making the later a more expensive procedure.^9^ Uptake of TPA biopsies is currently slower among patients from lower socioeconomic backgrounds and in regional hospitals.^7^ Within the TPA, there are 3 different ways to do it. The first (1) is using a mechanical arm where the ultrasound transducer (the same used for brachytherapy) is mounted along with a metal grid (transperineal grid biopsy [TP-Bx]), the second (2) is the needle-guided biopsy (Bx-GAG) where the transducer is not mounted on a mechanical arm, allowing for more freedom of movement, and a needle guide is attached, and finally, (3) the freehand transperineal prostate biopsy (fTP-Bx), where the transducer and needle are separate and can move independently.^9^ There is confusion in the literature regarding the terminology when discussing freehand, as many articles refer to “freehand” when the transducer is not mounted on a mechanical arm. However, technically, this is incorrect since “freehand” refers to not using any device other than the needle and transducer separately.^10-12^

Currently, most of the studies that exist on the TPA compare between TP-Bx and Bx-GAG, and there is a lack of publications specifically addressing the comparison between fTP-Bx and TP-Bx. Therefore, our main objective of this research is to fill this gap and delve into the comparative analysis of both methodologies.

## Material and Methods

After receiving approval from the Hospital Aleman of Buenos Aires ethics review board ((Approval no.: 202412, Date: 1-2-2024)), a retrospective review of a prospectively maintained database of all patients who consecutively underwent transperineal prostate biopsy (TPA biopsy) due to suspected prostate cancer (CaP) was conducted. This suspicion was based on elevated prostate-specific antigen (PSA) levels (≥4 ng/mL), a rectal examination suggestive of malignancy, or participation in an active surveillance protocol at our institution from December 2020 to June 2023. The biopsies were performed by 2 urologists, each with over 7 years of experience and having performed more than 200 procedures using the TPA.

Patients with a history of previous radiation treatment (e.g., external radiotherapy or brachytherapy) and those who underwent magnetic resonance imaging (MRI)/ultrasound (US) fusion biopsies were excluded.

Data on the demographic characteristics of the cohort, procedure specifics, and complications were prospectively collected and stored in a digital database. All procedures were performed under general anesthesia, and patients were discharged on the same day. Histopathological findings were reported using the Gleason Score in accordance with the International Society of Urological Pathology (ISUP). Complications were classified according to the Clavien–Dindo system.

### Magnetic Resonance Imaging

All multiparametric prostate MRIs were conducted using 1.5 T and 3 T MRI machines (GE Healthcare, Chicago, Illinois or Siemens Healthcare, Erlangen, Germany). Lesions were classified according to the Prostate Imaging Reporting and Data System (PI-RADS 2.0).^13^

### Biopsy Technique

**Figure 1.**
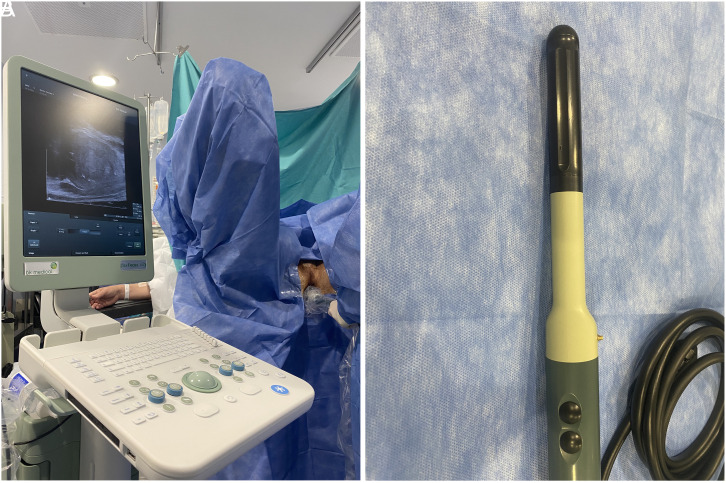
A, B. (A) High-resolution ultrasound machine (5000 BK, Cambridge MA, England), (B) in combination with an endocavitary transducer.

In all cases, an 18-Gauge Bard biopsy gun (Bard Biopsy Systems, Tempe, AZ) was used. A high-resolution ultrasound machine (5000 BK, Cambridge MA, England) was employed (Figure 1). Routine antibiotic prophylaxis with a first-generation cephalosporin was administered during anesthesia induction. No postoperative antibiotic treatment was prescribed.

### Grid Biopsy

**Figure 2.**
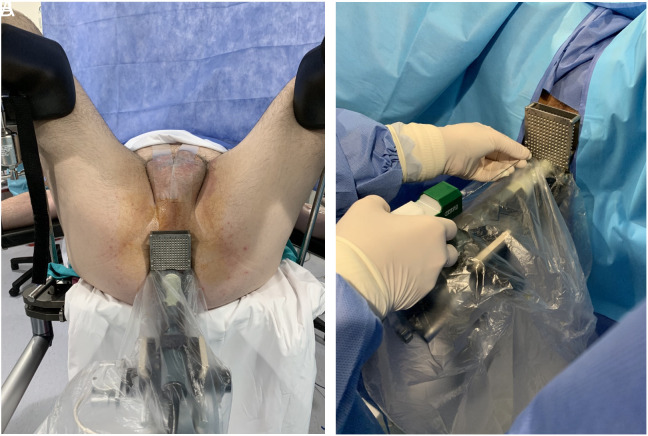
A, B. (A) The patient is positioned in a lithotomy forced position, with the scrotum elevated using adhesive tape to maximize exposure of the perineum. (B) A mechanical arm is used, onto which the ultrasound transducer is mounted, and a metal grid is attached adjacent to the perineum.

The patient is positioned in a lithotomy forced position, with the scrotum elevated using adhesive tape to maximize exposure of the perineum. A mechanical arm is used, onto which the ultrasound transducer is mounted, and a metal grid is attached adjacent to the perineum (Figure 2). Careful antisepsis with iodopovidone is performed over the entire perineal region before the placement of the endocavitary transducer (5000 BK, Cambridge MA, England). The recommended template for each lobe consists of 3 cores in the base, mid, and apical prostate zones, respectively, in the peripheral zone. Additionally, 2 cores are taken in the anterior region. In cases with a prior MRI showing target lesions, up to 3 cores are taken from each target lesion. The puncture is always monitored through sagittal and transverse scans using the biplanar transducer.

### Freehand Technique

**Figure 3.**
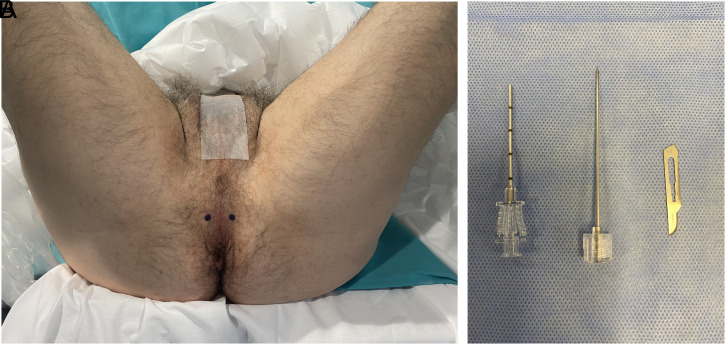
A, B. (A) Two access punctures are made, 1 for each prostate lobe, (B) using an 18-Gauge coaxial needle.

**Figure 4.**
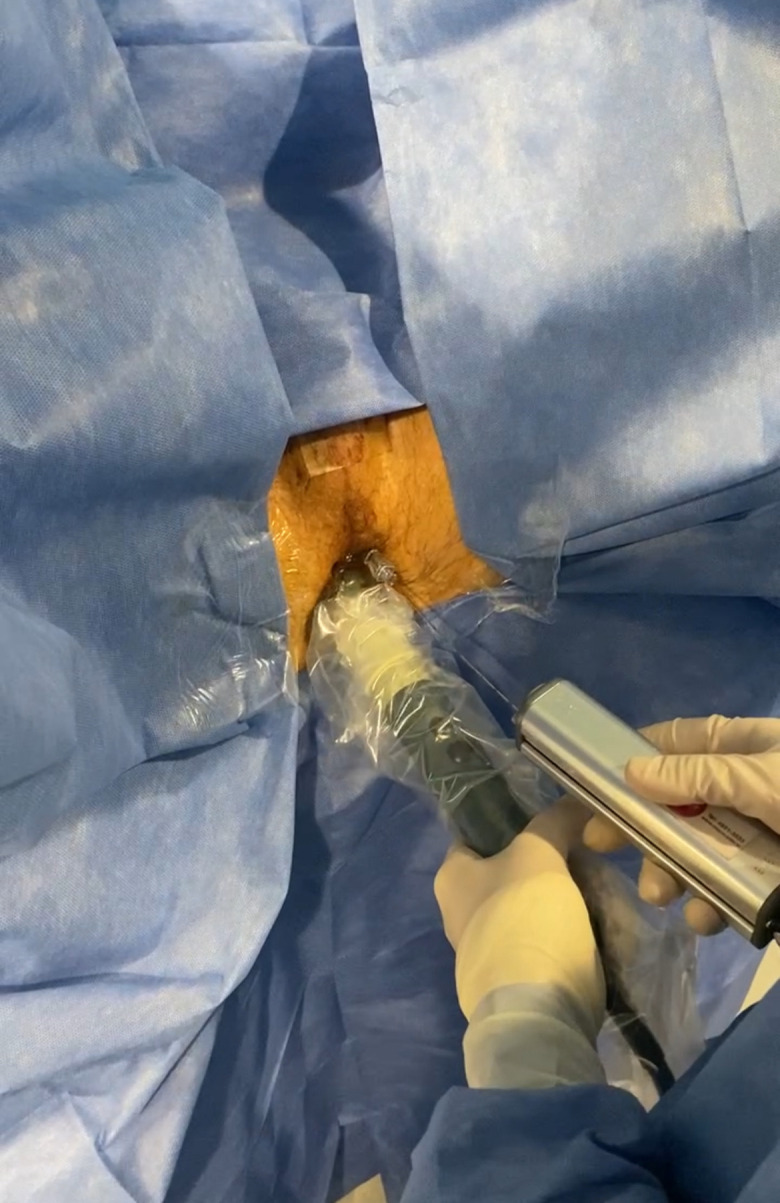
The patient is placed in a lithotomy position. The scrotum is then elevated using adhesive tape. Followed by the placement of the biplanar endocavitary transducer.

Here the patient is also placed in a forced lithotomy position, trying to expose the perineum as much as possible. Careful antisepsis with iodopovidone is carried out across the entire perineal region, followed by the placement of the biplanar endocavitary transducer. Two access punctures are made, 1 for each prostate lobe, using an 18-Gauge coaxial needle (Figures 3 and 4). At an ultrasound level, the prostatic urethra and the most lateral portion of the prostate were used as references, aiming to leave the coaxial needle at the midpoint. In the anteroposterior direction, the access should be directed towards the peripheral (posterior) zone of the prostate. The template for the peripheral zone involves taking 3 cores at the base, mid, and apical prostate levels in a “fan-like” method as described by Emiliozzi et al^14^ Regarding target lesions found in the MRI, the aim is not to take more than 3 cores from these lesions.

### Statistical Analysis

Clinically significant CaP was defined as ISUP 2 or higher. The chi-square test and Fisher’s exact test were used to compare categorical variables. For continuous variables the student *t*-test was used. Statistical significance was considered when *P* < .05.

### Population and Procedure Characteristics

A total of 533 patients comprised the initial population. After applying exclusion criteria, 89 patients were excluded: 24 due to a history of previous radiation treatment (including external radiotherapy and brachytherapy), and 65 for having undergone MRI/US fusion biopsies.

As a result, a total of 444 patients underwent transperineal biopsies (118 were TP-Bx, and 326 were fTP-Bx) (Table 1). The mean age was 67.9 years (standard deviation (SD) 8.8), and the entire population was of Hispanic origin. The mean PSA level was 12.6 ng/mL (SD 26.7), and the prostate volume was 54.5 cc (SD 27.9). Out of the total, 123 patients (27.7%) had a rectal examination suspicious of malignancy. The groups were comparable in PSA density (*P* = .35) and use of MRI (*P* = .20). From fTP-Bx group, the distribution of MRI results was as follows: 133 (63.44%) PIRADS 4-5, 40 (18.77%) PIRADS 3, and 40 (18.77%) PIRADS 2. In the TP-Bx group, the distribution was 42 (56.75%) PIRADS 4-5, 16 (21.62%) PIRADS 3, and 16 (21.62%) PIRADS 2. The percentage of patients on an active surveillance protocol was higher for fTP-Bx (*P* = .021).

### Cancer Detection

The average number of cores per biopsy was lower in those who underwent fTP-Bx (*P* < .0001). The mean number of cores taken per procedure in the fTP-Bx group was 20.1 (SD 5.5), while in the TP-Bx group it was 23.8 (SD 6.8). The overall detection rate for the study cohort was 70.1% (315/444).

To evaluate the detection rate further, the cohort was divided into patients with biopsy-naive (Bx-Naive) and patients with previous biopsies (Bx-No naive). The overall detection rate in the fTP-Bx group was higher than in the TP-Bx group, with no difference between Bx-Naive and Bx-No naive subgroups (*P* = .021). Of the total cohort, 64.6% (287/444) had undergone previous MRI.

The detection rate for clinically significant cancer (ISUP 2 or higher) was 53.8% (239/444). In the Bx-Naive group, it was 56.1% (197/351), with a statistically significant difference in the fTP-Bx group, where it was 59.7% (156/261) vs. 45.5% (41/90), *P* = .019. However, in the Bx-No naive group, it was 44.08% (41/93), with no significant difference between fTP-Bx (47.69%, 31/65) and TP-Bx (35.71%, 10/28), *P* = .28 (Table 2). A logistic regression analysis, including both univariate and multivariable analyses (Supplementary Table 1), indicated that freehand biopsy was associated with the detection of prostate cancer and clinically significant prostate cancer, after adjusting for age, PSA levels, and prostate volume.

### Complications

The total complication rate across both groups was 5.41% (24/444), with no statistically significant differences (Table 3). In the fTP-Bx group, there were no episodes of urinary infection confirmed by urine culture or hospitalization due to urological or other sources of sepsis. The most common complication was hematuria during the first 4 days. Only 1 patient received antibiotics for lower urinary tract symptoms despite a negative urine culture. In the fTP-Bx group, there were more episodes of acute urinary retention, with 3 cases compared to 1 in the TP-Bx group immediately after the procedure. One of these was due to hematuria, necessitating urethral catheter placement. In the TP-Bx group, 1 case of sepsis required hospitalization in an intermediate care unit.

## Discussion

Based on the results provided by our study, a statistically significant difference in favor of fTP-Bx regarding the detection rate of clinically significant cancer in the Bx-Naive group associated with the absence of infectious events compared to TP-Bx was observed. This may be due to multiple factors, mainly the possibility of puncturing any area of the prostate without having limitations or blind spots, a limitation present in the grid technique. All this is helped by having a good anatomical vision at an ultrasound level, both in longitudinal and transverse sections.

No studies specifically compared these 2 techniques, and this is partly due to the confusion that exists in the classification among different techniques within the same TPA. This is why, to have greater adherence to this type of technique, it is important to first have clarity on the nomenclature based on a classification, as mentioned by Ozden et al^15^ in his editorial. In fTP-Bx, the operator doesn’t need to angulate the transducer in the rectum; instead, angling solely the needle without any attachment to the transducer freely in the perineum is sufficient. This allows the operator to target any region of the prostate without excessive tilting of the transducer, which would increase patient discomfort. Regarding TP-Bx, according to our experience, it has certain disadvantages, the main ones being the presence of blind spots that the needle cannot reach due to the lower mobility of the needle and the need for multiple punctures in the perineal area, which would lead to supposedly greater post-procedure pain. As opposed, fTP-Bx also allows greater control of the needle trajectory and reduces unnecessary sampling of the transition zone. In fTP-Bx also has the added advantage of being less costly than TP-Bx, as it does not require the use of a mechanical arm and a metal grid.

The results published in the literature on the detection rate, although they compare different techniques, are variable.

Ristau et al^4^ showed a total detection of cancer and clinically significant cancer of 60.7% and 40.3%, respectively. Detection of any cancer (70.9% vs. 59.3%, *P* < .01) and clinically significant cancer (51.3% vs. 38.9%, *P* = .01) was higher using the Bx-GAG relative to the fTP-Bx. This increase in detection rate was greater using the Bx-GAG than fTP-Bx, partly due to the significant difference in the number of patients in each group (883 fTP-Bx vs. 117 Bx-GAG). Furthermore, the Bx-GAG group had a higher proportion of MRI (although the difference was not significant) and fewer patients under active surveillance.

Kasivisvanathan et al^16^ revealed a clinically significant cancer detection rate of 62% in 182 men with a median number of 40 cores. No major complications were reported. Our findings are similar to those obtained in the PROMIS study (overall and clinically significant rates of 71% and 57%, respectively, where the TPA was also used), but with fewer punctures performed.^17^ Additionally, our findings showed a higher rate than that reported by Szabo et al,^18^ whose study demonstrated overall and clinically significant prostate cancer detection rates of 43.4% (105/242) and 14% (35/242), respectively, using the Bx-GAG, with no significant difference to the control population undergoing fTP-Bx.

Complications were rare overall and the most severe complication (Clavien–Dindo 4) occurred in the TP-Bx group. In the fTP-Bx cohort, there were no cases of urinary infection confirmed by urine culture or the need for hospitalization due to sepsis. The most common complication was immediate postoperative hematuria (within 48 hours), which did not require treatment.

The limitations of our study include its retrospective design and single institution, which may limit generalizability to other populations. Additionally, the entire cohort consisted of Hispanic individuals, which limits generalization to other ethnic groups. Therefore, the number of cores was not standardized. Lastly, the number and location of cores could be influenced for those with prior multiparametric MRI, but there were no differences in the proportion of these patients between the 2 groups.

Our results show that within the TPA, the fTP-Bx offers a higher rate of overall and clinically significant cancer detection in biopsy-naive patients, with no infectious events.

## Supplementary Materials

Supplementary Material

## Figures and Tables

**Table 1.** Initial Demographic and Clinical Information

Parameter	Total (n: 444)	fTP-Bx (n: 326)	TP-Bx (n: 118)	*P*
Age	67.9 (SD 8.8)	67.6 (SD 8.9)	67.4 (SD 8.6)	.95
Prostate size (cc)	54.5 (SD 27.9)	55.8 (SD 28.7)	51.0 (SD 25.8)	.12
PSA (ng/mL)	12.6 (SD 26.7)	13.9 (SD 30.9)	9.2 (SD 7.7)	.11
PSA density (ng/mL)	0.26 (SD 0.64)	0.29 (SD 0.69)	0.21 (SD 0.22)	.35
MRI	287/444 (64.6%)	213 (63.4%)	74 (59.3%)	.17
PIRADS		2: 40 (18.77%) 3: 40 (18.77%) 4-5: 133 (65.3%)	2: 16 (21.62%) 3: 16 (21.62%) 4-5: 42 (56.7%)	
Mean number of cores	21.1 (SD 6.1)	20.1 (SD 5.5)	23.8 (SD 6.8)	<.0001*
Active surveillance	35 (7.8%)	22 (6.7%)	13 (7.39%)	.14
Previous biopsy		65/326 (20%)	28/118 (23.7%)	.39

Values are presented as mean (SD) for continuous variables, and the number of patients (percentage) for categorical variables.

fTP-Bx, freehand transperineal prostate biopsy; MRI, magnetic resonance imaging; TP-Bx, transperineal grid biopsy; PIRADS, prostate imaging reporting and data system; PSA, prostate-specific antigen.

**Table 2.** Overall and Clinically Significant Cancer Detection

Parameter	Total Cohort	fTP-Bx (n: 326)	TP-Bx (n: 118)	*P*
Overall Ca	315/444 (70.1%)	241/326 (73.9%)	74/118 (62.7%)	.021*
ISUP 2 or higher	239/444 (53.8%)	188/326 (57.6%)	51/118 (43.2%)	.006
ISUP 2 or higher—Bx naive	197/351 (56.1%)	156/261 (59.7%)	41/90 (45.5%)	.019
ISUP 2 or higher—Bx no naive	41/93 (44.08%)	31/65 (47.69%)	10/28 (35.71)	.28

ISUP refers to the International Society of Urological Pathology Grade, where ISUP 2 or higher indicates clinically significant cancer.

Bx, biopsy; fTP-Bx, freehand transperineal prostate biopsy; ISUP, international society of urological pathology; TP-Bx, transperineal grid biopsy.

**Table 3.** Complications According to Clavien–Dindo

Parameter	Total	fTP-Bx (n: 326)	TP-Bx (n: 118)	*P*
Complications	24/444 (5.41%)	17/326 (5.2%)	7/118 (5%)	.76
Clavien 1	17 (70.83%)	14 (82.35%)	3 (17.64%)	
Clavien 2	6 (25%)	3 (50%)	3 (50%)	
Clavien 3	0 (0%)	0 (0%)	0 (0%)	
Clavien 4	1 (4.16%)	0 (0%)	1 (100%)	

Clavien–Dindo classification includes Clavien 1 (minor complications), Clavien 2 (complications requiring pharmacological treatment, blood transfusion, or parenteral nutrition), Clavien 3 (complications requiring surgical, endoscopic, or radiological intervention), and Clavien 4 (life-threatening complications).

fTP-Bx, freehand transperineal biopsy; TP-Bx, transperineal grid biopsy.

**Supplementary Table 1.** Univariate and Multivariate Logistic Regression Analyses for Cancer and Clinically Significant Cancer Detection

	Prostate Cancer						Clinically Significant Prostate Cancer					
	Univariate			Multivariate			Univariate			Multivariate		
	OR	95% CI	*P*	OR 95% CI	*P*		OR	95% CI	*P*	OR	95% CI	*P*
Biopsy approach												
No free hand	ref	-	-	ref	-	-	ref	-	-	ref	-	-
Free hand	1.67	1.06-2.61	.026	1.69	1.03-3.78	0.036	1.75	1.14-2.69	.01	1.86	1.16-2.96	.01
Protate volume, cc												
≤30	ref	-	-	ref	-	-	ref	-	-	ref	-	-
>30	0.5	0.27-0.96	.036	0.46-0.23-0.93	.031		0.49	0.29-0.85	.01	0.5	0.27-0.90	.022
PSA, ng/mL												
≤10	ref	-	-	ref	-	-	ref	-	-	ref	-	-
>10	1.59	1.01-2.49	.042	1.56	.95-2.53	0.075	1.55	1.04-2.29	.03	1.38	0.89-2.12	.16
Age, years												
≤70	ref	-	-	ref	-	-	ref	-	-	ref	-	-
>70	3.51	2.17-5.69	<.001	3.51	2.11-5.8	<0.001	3.53	2.34-5.34	<.001	3.56	2.30-5.48	<.001

CI, confidence interval; OD, odds ratio; PSA, prostate-specific antigen.

## Data Availability

The data of this study is available upon reasonable request to the corresponding author.

## References

[1] Takenaka A Hara R Ishimura T , et al. A prospective randomized comparison of diagnostic efficacy between transperineal and transrectal 12-core prostate biopsy. Prostate Cancer Prostatic Dis. 2008;11(2):134 138. (10.1038/sj.pcan.4500985) 17533394

[2] Abdollah F Novara G Briganti A , et al. Trans-rectal versus trans-perineal saturation rebiopsy of the prostate: is there a difference in cancer detection rate?. Urology. 2011;77(4):921 925. (10.1016/j.urology.2010.08.048) 21131034

[3] Huang GL Kang CH Lee WC Chiang PH . Comparisons of cancer detection rate and complications between transrectal and transperineal prostate biopsy approaches - a single center preliminary study. BMC Urol. 2019;19(1):101. (10.1186/s12894-019-0539-4) 31660936 PMC6816188

[4] Ristau BT Allaway M Cendo D , et al. Free-hand transperineal prostate biopsy provides acceptable cancer detection and minimizes risk of infection: evolving experience with a 10-sector template. Urol Oncol. 2018;36(12):528.e15 528.e20. (10.1016/j.urolonc.2018.09.013) 30446447

[5] Pepe P Aragona F . Morbidity after transperineal prostate biopsy in 3000 patients undergoing 12 vs 18 vs more than 24 needle cores. Urology. 2013;81(6):1142 1146. (10.1016/j.urology.2013.02.019) 23726443

[6] Grummet JP Weerakoon M Huang S , et al. Sepsis and ‘superbugs’: should we favour the transperineal over the transrectal approach for prostate biopsy?. BJU Int. 2014;114(3):384 388. (10.1111/bju.12536) 24612341

[7] Zattoni F Rajwa P Miszczyk M , et al. Transperineal versus transrectal magnetic resonance imaging-targeted prostate biopsy: a systematic review and meta-analysis of prospective studies. Eur Urol Oncol. 2024;7(6):1303 1312. (10.1016/j.euo.2024.07.009) 39095298

[8] Miah S Eldred-Evans D Simmons LAM , et al. Patient reported outcome measures for transperineal template prostate mapping biopsies in the picture study. J Urol. 2018;200(6):1235 1240. (10.1016/j.juro.2018.06.033) 29940251

[9] Berry B Parry MG Sujenthiran A , et al. Comparison of complications after transrectal and transperineal prostate biopsy: a national population-based study. BJU Int. 2020;126(1):97 103. (10.1111/bju.15039) 32124525

[10] Urkmez A Demirel C Altok M Bathala TK Shapiro DD Davis JW . Freehand versus Grid-Based transperineal prostate biopsy: a comparison of anatomical region yield and complications. J Urol. 2021;206(4):894 902. (10.1097/JU.0000000000001902) 34100650

[11] Setia SA Smith J Cendo D , et al. Outcomes of freehand transperineal prostate biopsy with omission of antibiotic prophylaxis. BJU Int. 2022;130(1):54 61. (10.1111/bju.15590) 34491606

[12] Wilcox Vanden Berg RN George AK Kaye DR . Should transperineal prostate biopsy be the standard of care?. Curr Urol Rep. 2023;24(3):135 142. (10.1007/s11934-022-01139-0) 36512186

[13] Turkbey B Choyke PL . PIRADS 2.0: what is new?. Diagn Interv Radiol. 2015;21(5):382 384. (10.5152/dir.2015.15099) 26200484 PMC4557320

[14] Emiliozzi P Longhi S Scarpone P Pansadoro A DePaula F Pansadoro V . The value of a single biopsy with 12 transperineal cores for detecting prostate cancer in patients with elevated prostate specific antigen. J Urol. 2001;166(3):845 850. (10.1016/S0022-5347(05)65849-1) 11490231

[15] Özden E Öztürk E Turgut AT . Freehand versus grid-based transperineal prostate biopsy: a comparison of anatomical region yield and complications. Letter. J Urol. 2023;210(2):248 249. (10.1097/JU.0000000000003546) 37163219

[16] Kasivisvanathan V Rannikko AS Borghi M , et al. MRI-targeted or standard biopsy for prostate-cancer diagnosis. N Engl J Med. 2018;378(19):1767 1777. (10.1056/NEJMoa1801993) 29552975 PMC9084630

[17] Ahmed HU El-Shater Bosaily A Brown LC , et al. Diagnostic accuracy of multi-parametric MRI and TRUS biopsy in prostate cancer (PROMIS): a paired validating confirmatory study. Lancet. 2017;389(10071):815 822. (10.1016/S0140-6736(16)32401-1) 28110982

[18] Szabo RJ . Free-hand transperineal prostate biopsy under local anesthesia in the office without antibiotic prophylaxis: experience with 304 cases. J Endourol. 2021;35(4):518 524. (10.1089/end.2020.1086) 33573475

