# Supplementary material for: Freehand Transperineal Prostate Biopsy Improves the Detection Rate of Clinically Significant Prostate Cancer
Source: Urol Res Pract. 2025 Mar 6;50(5):269–74. doi: 10.5152/tud.2025.24026 (PMC11923604; doi:10.5152/tud.2025.24026)
Supplement: Supplementary Material [file supplementary_material.pdf]

Supplementary Table 1. Univariate and Multivariate Logistic Regression Analyses for Cancer and Clinically Significant Cancer Detection

	Prostate Cancer						Clinically Significant Prostate Cancer					
	Univariate			Multivariate			Univariate			Multivariate		
	**OR**	**95% CI**	***P***	**OR 95% CI**	***P***		**OR**	**95% CI**	***P***	**OR**	**95% CI**	***P***
Biopsy approach												
No free hand	ref	-	-	ref	-	-	ref	-	-	ref	-	-
Free hand	1.67	1.06-2.61	.026	1.69	1.03-3.78	0.036	1.75	1.14-2.69	.01	1.86	1.16-2.96	.01
Protate volume, cc												
≤30	ref	-	-	ref	-	-	ref	-	-	ref	-	-
>30	0.5	0.27-0.96	.036	0.46-0.23-0.93	.031		0.49	0.29-0.85	.01	0.5	0.27-0.90	.022
PSA, ng/mL												
≤10	ref	-	-	ref	-	-	ref	-	-	ref	-	-
>10	1.59	1.01-2.49	.042	1.56	.95-2.53	0.075	1.55	1.04-2.29	.03	1.38	0.89-2.12	.16
Age, years												
≤70	ref	-	-	ref	-	-	ref	-	-	ref	-	-
>70	3.51	2.17-5.69	<.001	3.51	2.11-5.8	<0.001	3.53	2.34-5.34	<.001	3.56	2.30-5.48	<.001

CI, confidence interval; OD, odds ratio; PSA, prostate-specific antigen.
